# A Role for TGFβ Signaling in Preclinical Osteolytic Estrogen Receptor-Positive Breast Cancer Bone Metastases Progression

**DOI:** 10.3390/ijms22094463

**Published:** 2021-04-24

**Authors:** Julia N. Cheng, Jennifer B. Frye, Susan A. Whitman, Andrew G. Kunihiro, Ritu Pandey, Janet L. Funk

**Affiliations:** 1 Cancer Biology Graduate Interdisciplinary Program, University of Arizona, Tucson, AZ 85724, USA; juliacheng@email.arizona.edu; 2 Department of Medicine, University of Arizona, Tucson, AZ 85724, USA; jabeisch@email.arizona.edu (J.B.F.); susanwhitman@email.arizona.edu (S.A.W.); 3 Department of Nutritional Sciences, University of Arizona, Tucson, AZ 85724, USA; akunihiro@email.arizona.edu; 4 Department of Cellular and Molecular Medicine, University of Arizona, Tucson, AZ 85724, USA; ritu@arizona.edu

**Keywords:** breast cancer, bone metastases, estrogen receptor positive, TGFβ, PTHrP

## Abstract

While tumoral Smad-mediated transforming growth factor β (TGFβ) signaling drives osteolytic estrogen receptor α-negative (ER-) breast cancer bone metastases (BMETs) in preclinical models, its role in ER+ BMETs, representing the majority of clinical BMETs, has not been documented. Experiments were undertaken to examine Smad-mediated TGFβ signaling in human ER+ cells and bone-tropic behavior following intracardiac inoculation of estrogen (E_2_)-supplemented female nude mice. While all ER+ tumor cells tested (ZR-75-1, T47D, and MCF-7-derived) expressed TGFβ receptors II and I, only cells with TGFβ-inducible Smad signaling (MCF-7) formed osteolytic BMETs in vivo. Regulated secretion of PTHrP, an osteolytic factor expressed in >90% of clinical BMETs, also tracked with osteolytic potential; TGFβ and E_2_ each induced PTHrP in bone-tropic or BMET-derived MCF-7 cells, with the combination yielding additive effects, while in cells not forming BMETs, PTHrP was not induced. In vivo treatment with 1D11, a pan-TGFβ neutralizing antibody, significantly decreased osteolytic ER+ BMETs in association with a decrease in bone-resorbing osteoclasts at the tumor-bone interface. Thus, TGFβ may also be a driver of ER+ BMET osteolysis. Moreover, additive pro-osteolytic effects of tumoral E_2_ and TGFβ signaling could at least partially explain the greater propensity for ER+ tumors to form BMETs, which are primarily osteolytic.

## 1. Introduction

The majority of patients with metastatic breast cancer have bone metastases (BMETs), which are primarily osteolytic and currently lack a cure [1,2,3,4]. Most BMETs occur in patients with estrogen receptor-positive (ER+) tumors, both due to the higher prevalence of this tumor subtype, including among those with metastatic disease (70%), as well as the higher prevalence of BMETs in ER+ (vs. ER-) metastatic disease [5,6,7,8]. Despite rates of initial tumor cell dissemination to bone that appear similar regardless of ER status, clinically evident BMETs develop with twice the frequency in ER+ (vs. ER-) metastatic disease and remain concordant for ER+ expression in a majority of cases [9,10,11]. In contrast, ER+ metastasis formation rates at non-bone sites are less than or equal to those of ER- tumors [8]. Therefore, specific modeling of ER+ breast cancer BMET is clinically relevant due to its prevalence, and also due to the possibility that tumoral ER+ signaling could be a mediator, and not just a marker, specific to metastasis progression within the bone microenvironment. Recent experiments conducted by our laboratory support this postulate, providing what is, to the best of our knowledge, the first evidence of a possible causal role for tumoral ER signaling in mediating tumor-associated osteolysis in ER+ BMET [12]. In vivo peritumoral osteolysis, osteoclast formation, and osteolytic BMET progression all progressed in an estrogen (E_2_) dose-dependent fashion in a human ER+ breast cancer cell (MCF-7) osteolytic BMET model, independent of direct estrogenic effects on the bone milieu or tumor cell proliferation, but in association with ER-mediated tumoral secretion of parathyroid hormone-related protein (PTHrP), an osteolytic factor expressed with greater prevalence in osseus vs. non-osseus metastases [13,14,15,16,17,18], providing a possible mechanistic basis for the E_2_-driven tumor-associated osteolysis documented in vivo.

In addition to its common expression in clinical BMET, PTHrP has been a molecule of interest in the pathogenesis of osteolytic breast cancer BMET since being identified as a primary driver of metastatic progression in the first preclinical breast cancer BMET model described over two decades ago, which utilized bone tropic ER- MDA-MB-231 cells, variants of which have remained a mainstay of preclinical BMET research to the present day [14,15,19,20]. A large body of evidence now exists, using MDA-MB-231 variants and other ER- cells (e.g., murine 4T1), that identifies tumoral Smad-mediated transforming growth factor β (TGFβ) signaling as a primary driver of osteolytic BMET progression in ER- BMET models [19,21,22,23]. TGFβ released from resorbed bone matrix mediates these effects via binding to tumor cell TGFβ receptor II (TGFβRII), activating canonical Smad2/3 signaling with additional contributions of non-canonical TGFβ signaling pathways (e.g., mitogen-activated protein kinases [MAPK]), subsequently inducing tumoral expression of pro-metastatic factors [23,24,25,26], including PTHrP, the same osteolytic factor induced by E_2_ in the ER+ BMET model. In ER- models, specific blockade of tumoral Smad-mediated TGFβ signaling [22,24,27,28] reduces osteolytic ER- BMET size in combination with a reduction in tumor-associated osteoclasts, thus revealing a key pathogenic role for tumoral TGFβ signaling in osteolysis. An important but potentially more limited role for TGFβ signaling in osteoclast precursors in metastatic progression in these same models has been identified [29,30]. Untangling the complexities of tumoral vs. bone TGFβ signaling is difficult. However, therapeutic benefits of TGFβ neutralization are clear in ER- BMET models, as numerous studies have demonstrated significant reductions in osteolytic ER- BMET progression in response to systemic neutralization of TGFβ signaling [31,32], leading to a proposed use of TGFβ-targeting therapeutics in breast cancer BMETs treatment [21,33].

Still largely unexplored, however, is whether TGFβ, and in particular tumoral TGFβ signaling, has a role in driving bone metastatic progression for ER+ tumors. This is a relevant question since in vitro evidence in ER+ human breast cancer cells of reciprocal expression of ERα and TGFβRII [34,35,36] and E_2_ inhibition of TGFβ-induced Smad signaling [37,38,39,40] suggests that anti-TGFβ therapeutics could be less effective for the majority of patients with BMETs, i.e., those with ER+ tumors, if tumoral TGFβ signaling in ER+ cells does not contribute to osteolytic BMET progression. Therefore, studies were undertaken to examine a role for TGFβ signaling in ER+ osteolytic BMET progression using a variety of in vivo human ER+ osteolytic breast cancer xenograft models, including an assessment of tumoral Smad-mediated TGFβ signaling and possible crosstalk in bone tropic ER+ breast cancer cells between osteolytic TGFβ and E_2_ signaling pathways.

## 2. Results

### 2.1. TGFβR Expression and TGFβ-Stimulated Smad Signaling in Estrogen Receptor-Positive (ER+) Breast Cancer Cells

**Figure 1:**
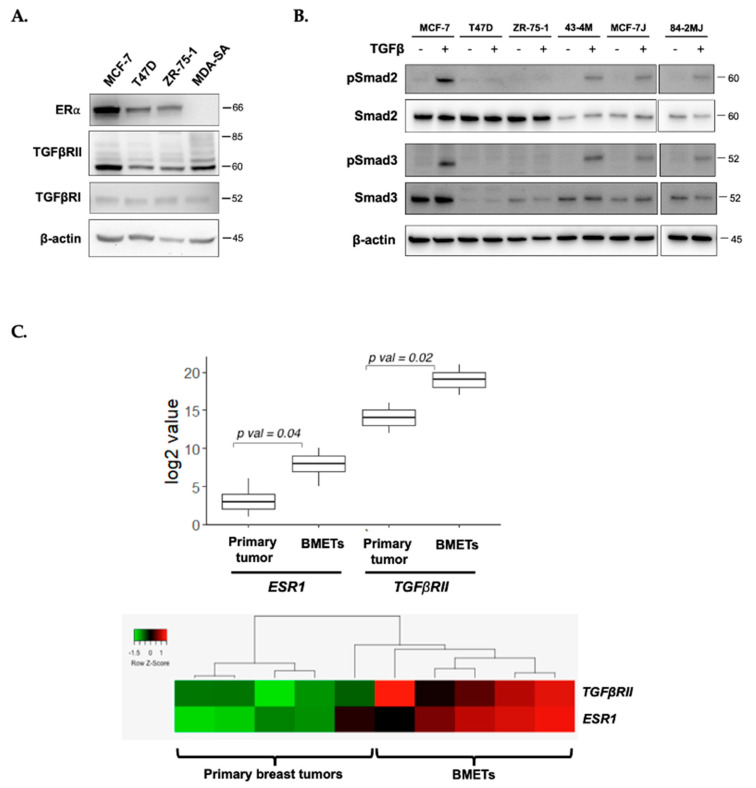
Transforming growth factor β (TGFβ) receptor expression and TGFβ-inducible Smad signaling in estrogen receptor-positive (ER+) breast cancer cells. (**A**) Constitutive ERα, TGFβRII, and TGFβRI protein levels in human ER+ MCF-7, T47D, and ZR-75-1 cells as compared with ER- MDA-SA cells, by Western blot analysis; (**B**) Smad2/3 expression and TGFβ-stimulated (5 ng/mL for 1 h) Smad2 and Smad3 phosphorylation in MCF-7, MCF-7J, T47D, ZR-75-1, and bone metastasis (BMET)-derived 43-4M (from MCF-7) and 84-2MJ (from MCF-7J) cells, from a single blot; (**C**) elevated (top) co-expression (bottom) of *ESR1* and *TGFβRII* genes in human clinical BMETs vs. primary breast tumors from GEO dataset GSE39494 (*n* = 5/group, unpaired).

Luminal ERα-expressing (ER+) human breast cancer cell lines, MCF-7, T47D, and ZR-75-1 (Figure 1A), each expressed TGFβ receptors II and I (TGFβRII and I, Figure 1A) at levels similar to those in bone-tropic MDA-MB-231 breast cancer cells (MDA-SA), an ER- cell line provided by Dr. Theresa Guise that forms TGFβ-dependent BMET in vivo [22,24,41]. However, Smad signaling (phosphorylation of Smad2 or Smad 3) was only TGFβ-inducible in MCF-7 cells, and not in T47D or ZR-75-1 cells (Figure 1B). Notably, while Smad2 and Smad4, a required co-factor for pSmad2/3-mediated gene expression (Supplemental Figure S1), were expressed in all ER+ cell lines, the lack of Smad3 phosphorylation in T47D and ZR-75-1 cells was associated with markedly lower or undetectable Smad3 levels (Figure 1B).

Consistent with the findings of ER and TGFβRII co-expression in human ER+ breast cancer cell lines, in a clinical breast cancer series, *ESR1* and *TGFβRII* genes were similarly co-expressed in breast cancer primary tumors and BMETs (Figure 1C). Notably, expression levels of both receptors were significantly higher in tumor cells derived from BMETs, as compared with cells in primary tumors from women with BMETs (Figure 1C).

### 2.2. Only ER+ Cells with TGFβ-Inducible Smad Signaling Formed Osteolytic Bone Metastases (BMETs) In Vivo

**Figure 2:**
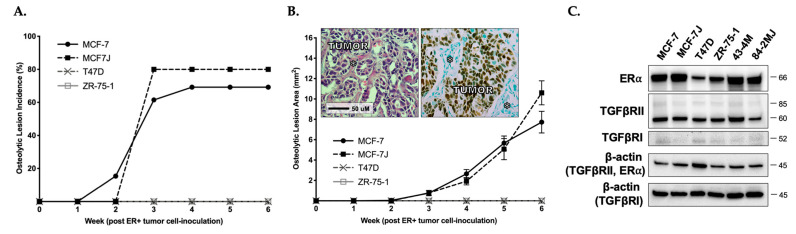
In vivo osteolytic BMET progression of ER+ breast cancer cells inoculated into E_2_-supplemented mice. (**A**) Osteolytic BMET lesion incidence and (**B**) osteolytic lesion area in hind limbs of E_2_-supplemented mice inoculated with ER+ tumor cells as measured by radiographs and confirmed by histology (*n* = 5–13/group). Mice injected with T47D or ZR-75-1 cells did not form any visibly detectable osteolytic BMET lesions. Inset, representative histological cross-sections of metastases in bone that were H&E- (left) or ERα immuno-stained (right, brown) demonstrate luminal structure and ERα expression of ER+ MCF-7 BMETs. *, bone. (**C**) ERα, TGFβRII, and TGFβRI protein expression, as demonstrated by Western blot, in ER+ MCF-7, MCF-7J, T47D, or ZR-75-1 cells as compared with BMET-derived 43-4M (from MCF-7) or 84-2MJ (from MCF-7J) cells. Results are representative of findings in additional MCF-7-BMET-derived cells tested (data not shown).

When MCF-7 cells, MCF-7J (a MCF-7 subline transfected to express luciferase), T47D, or ZR-75-1 cells were inoculated into E_2_-supplemented female athymic nude mice, using methods previously established [12,41], only ER+ cells with TGFβ-inducible Smad signaling (MCF-7 and MCF-7J, Figure 1B) formed osteolytic BMETs. Osteolytic BMET lesions, confirmed by histology (MCF-7) or BLI (MCF-7J), reached maximal incidence by 3 weeks post tumor inoculation and were still increasing in size by the study’s end at 6 weeks (Figure 2A,B). Tumor cells within BMETs retained their luminal-type structure (Figure 2B inset, left) and ERα expression (Figure 2B inset, right, brown), and tumor cells isolated from these ER+ BMETs (43-4M from MCF-7-inoculated and 84-2MJ from MCF-7J-inoculated) retained the characteristics of inoculated cells with regards to TGFβ-inducible Smad signaling, albeit with somewhat reduced levels of Smad expression (Figure 1B); ER, TGFβRII, and TGFβRI receptor expression (Figure 2C); and epithelial phenotype (expressing E-cadherin, but not vimentin, contrasting with ER- MDA-SA cells, Supplemental Figure S2). Notable was the absence of non-osseous metastases in mice inoculated with MCF-7 or MCF-7J cells, as determined by gross necropsy or bioluminescence.

In contrast to MCF-7 and MCF-7J cells, T47D and ZR-75-1 cells did not form osteolytic BMETs in vivo (Figure 2A,B), nor was there any histological evidence of ER+ tumors in hind limb cross sections (data not shown). Interestingly, however, ER+ human tumor cells could still be isolated and propagated from hind limbs of E_2_-supplemented T47D and ZR-75-1-inoculated mice 6 weeks post inoculation, albeit with a lower frequency as compared with mice with MCF-7-derived BMETs (e.g., 33% of hind limbs from ZR-75-inoculated vs. 74% from MCF-7-inoculated), suggesting that the complete lack of osteolytic BMET formation in T47D and ZR-75-1-inoculated mice was not due to the absence of tumor cell dissemination to bone. Differences in proliferation rates among the cell lines also could not explain the differential appearance of osteolytic lesions in MCF-7-inoculated mice at 2 weeks vs. complete absence of lesions after 6 weeks (or up to 9 weeks in a small number of mice (data not shown)) in T47D- or ZR-75-1-inoculated mice, since in vitro doubling times for T47D or ZR-75-1 cells in estrogen-replete media were similar to, or only two-fold longer, than MCF-7 cells (48, 54, and 91 h, for MCF-7, T47D, and ZR-75-1, respectively).

### 2.3. Effects of E_2_ and/or TGFβ on ERα, TGFβRII, and Smad Activation in Bone-Tropic ER+ Cells

**Figure 3:**
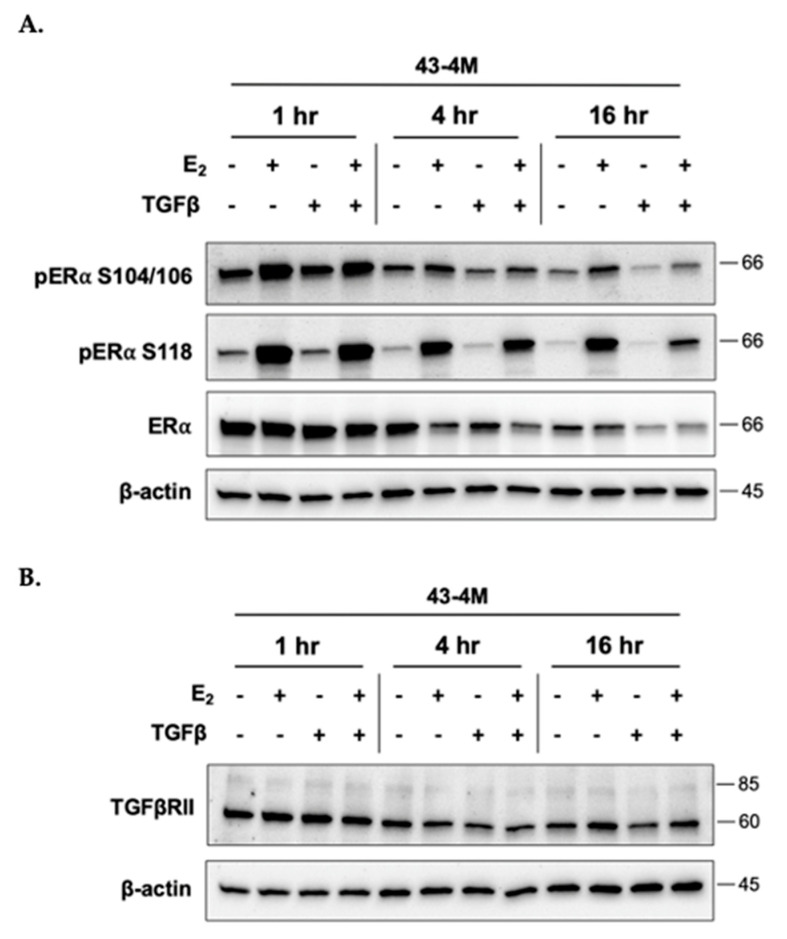
Effects of E_2_ and TGFβ on ERα activation and TGFβRII expression of BMET-derived ER+ breast cancer cells. (**A**) Constitutive and E_2_-stimulated (S104/106 and S118 phosphorylated) ERα and (**B**) TGFβRII expression in 43-4M cells maintained in E_2_-deplete media for 4 days prior to simultaneous treatment with E_2_ (10^−8^ M) and/or TGFβ (5 ng/mL) for the indicated times.

**Figure 4:**
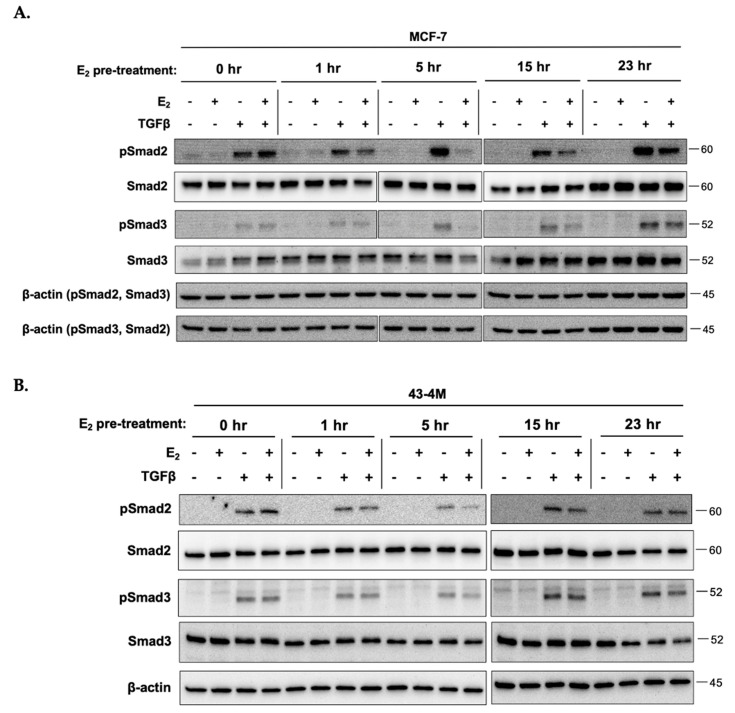
E_2_ transiently decreases TGFβ-induced Smad signaling in bone-tropic ER+ breast cancer cells. Smad2/3 expression and TGFβ-stimulated (5 ng/mL for 1 h) Smad2 and Smad3 phosphorylation in (**A**) MCF-7 and (**B**) MCF-7 BMET-derived 43-4M cells maintained in E_2_-deplete media for 4 days prior to stimulation with E_2_ (10^−8^ M) vs. E_2_-deplete media for the indicated times prior to addition of TGFβ (5 ng/mL) for one hour. Note, early (0–5 h) vs. late times (15–23 h) were run on two separate blots.

Due to reports of antagonistic crosstalk between E_2_ and Smad-mediated TGFβ signaling in breast cancer cells [34,35,37,38,39,40] and our previous demonstration of the E_2_-dependency of osteolysis in ER+ MCF-7 BMETs [12], the time dependent effects of each agent on expression and/or activation of the opposing receptor were assessed in bone-tropic ER+ tumor cells. Untreated control cells were included at each time point to account for possible changes in protein levels attributable to the addition of fresh media at the start of the experiment (Figure 3A). TGFβ stimulation of MCF-7 BMET-derived ER+ 43-4M cells, although tending to reduce ERα levels at the later times, did not prevent E_2_ activation of ERα (i.e., phosphorylation at S104/106 and S118) at any time (Figure 3A). Furthermore, TGFβ stimulation alone did not result in ERα phosphorylation (Figure 3A), a putative mechanism by which growth factors can alter ER signaling [42]. E_2_ stimulation did not reduce TGFβRII levels (Figure 3B), indeed, at later timepoints, TGFβRII levels were slightly increased in E_2_-treated cells. When assessing E_2_ effects on TGFβ-induced Smad signaling in MCF-7 (Figure 4A) and MCF-7 BMET-derived 43-4M (Figure 4B) cells, E_2_ pretreatment downregulated TGFβ-induced Smad activation in both cell lines, an effect that peaked with 5 h of E_2_ pretreatment; however, this effect was transient and of much shorter duration in BMET-derived ER+ 43-4M cells. Thus, neither E_2_ nor TGFβ appeared to prevent the expression or activation of the opposing receptor, including TGFβ-mediated Smad signaling, in ER+ cells isolated from osteolytic BMETs.

### 2.4. Effects of E_2_ and/or TGFβ on Pro-Metastatic PTHrP Secretion from ER+ Cells

**Figure 5:**
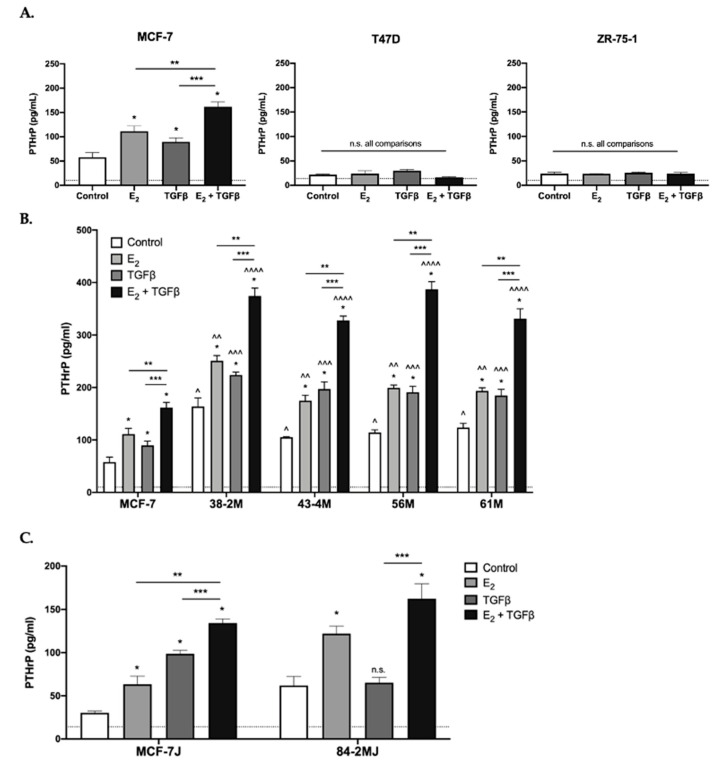
E_2_ and/or TGFβ-inducible tumor secretion of osteolytic factor PTHrP. Osteolytic PTHrP secretion from (**A**) ER+ MCF-7, T47D, or ZR-75-1 cells; (**B**) MCF-7 vs. MCF-7 BMET-derived ER+ tumor cells (38-2M, 43-4M, 56M, 61M); (**C**) MCF-7J vs. MCF-7J BMET-derived (84-2MJ) tumor cells. Cell were estrogen-depleted prior to stimulation with E_2_ (10^−7^ M) and/or TGFβ (5 ng/mL) vs. E_2_-deplete media alone for 48 (MCF-7, MCF-7-derived, and ZR-75-1 cells) or 72 h (T47D, MCF-7J, and 84-2MJ) (*n* = 3–4/group). Cell number (by MTT assay) was not altered by E_2_ or TGFβ treatment under the conditions of the experiment (data not shown). * *p ≤* 0.05 vs. media control, ** *p ≤* 0.05 E_2_ vs. E_2_ + TGFβ, *** *p ≤* 0.05 TGFβ vs. E_2_ + TGFβ, or not significant (n.s.), as measured by one-way ANOVA with Holm–Sidak’s post hoc test. ^ *p ≤* 0.05 vs. MCF-7 control, ^^ *p ≤* 0.01 vs. MCF-7 with E_2_, ^^^ *p ≤* 0.0001 vs. MCF-7 with TGFβ, ^^^^ *p ≤* 0.0001 vs. MCF-7 with E_2_ + TGFβ, as measured by two-way ANOVA with Dunnett’s post hoc test. Assay sensitivities are indicated by dotted lines.

PTHrP secretion, which we previously demonstrated to be E_2_ inducible via ERα in MCF-7 cells [12] and is TGFβ inducible via Smad signaling in some bone-tropic ER- breast cancer cell lines [13,14,15,43], was also induced by TGFβ in bone-tropic MCF-7 cells, demonstrating additive effects when combined with E_2_ (Figure 5A, left panel). However, in ER+ cells that did not form osteolytic BMETs (T47D and ZR-75-1), PTHrP levels were near or at the limit of detection and were not altered by E_2_ or TGFβ, alone or in combination (Figure 5A, middle and right panels). In ER+ cells isolated from ER+ BMETs (38-2M, 43-4M, 56M, and 61M derived from MCF-7 cells, or 84-2MJ derived from MCF-7J cells), PTHrP secretion remained E_2_ and TGFβ inducible, with additive effects in combination (Figure 5B,C). Notably, in ER+ cells isolated from BMETs, PTHrP secretion (constitutive or stimulated in response to E_2_, TGFβ, and/or the combination) was higher as compared with MCF-7 or MCF-7J tumor cells initially inoculated (Figure 5B,C).

### 2.5. Effect of E_2_ and/or TGFβ on Non-Smad Signaling Pathways in Bone-Tropic ER+ Cells

**Figure 6:**
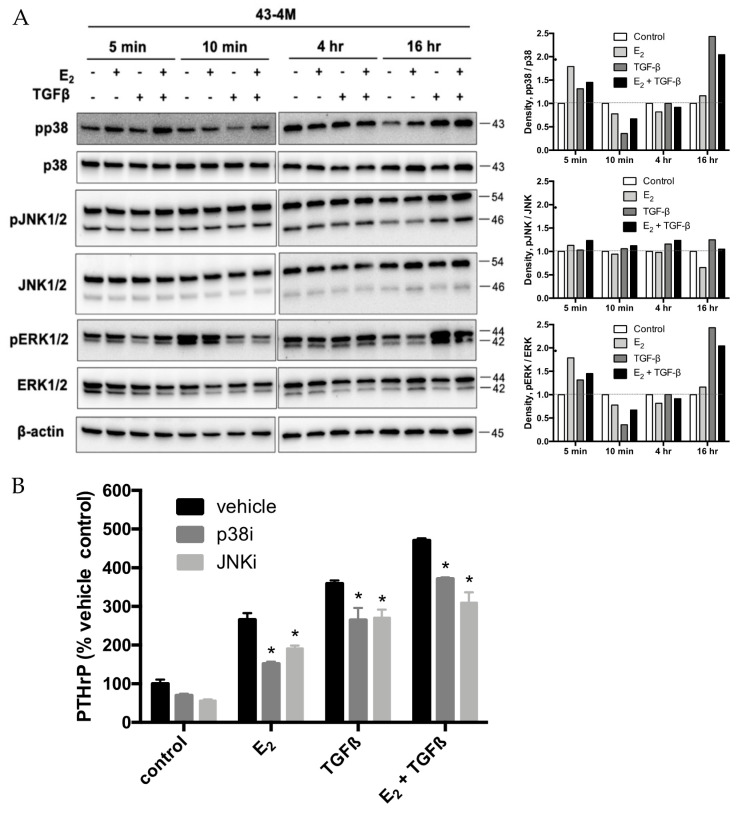
Effect of E_2_ and/or TGFβ on MAPK signaling. (**A**) Western analysis of MAPK (p38, ERK1/2, and JNK1/2) protein expression and activation (phosphorylation) in 43-4M cells maintained for 4 days in E_2_-deplete media prior to concurrent treatment with E_2_ (10^−8^ M) and/or TGFβ (5 ng/mL) for indicated times (left panel). Note, early (5–10 min) vs. late times (4–16 h) were run on two separate blots. Densitometric analysis of MAPK phosphorylation levels relative to MAPK protein levels, normalized to control for each time point, is also included (right panel); (**B**) inducible PTHrP secretion in 43-4M. M cells maintained for 4 days in E_2_-deplete media prior to 52 h of treatment with E_2_ (10^−8^ M) and/or TGFβ (5 ng/mL) vs. media control, with or without 1 h pretreatment with p38 inhibitor SB202190 (10 µM) or JNK inhibitor SP600125 (25 µM) vs. vehicle (*n* = 4–8/group). * *p ≤* 0.01, inhibitor vs. vehicle with E_2_ and/or TGFβ treatment by two-way ANOVA with Sidak’s post hoc test. Inhibitors significantly decreased constitutive secretion (see controls) by *t* test (*p* < 0.05), but not by ANOVA. When re-expressed as % change relative to constitutive levels, no inhibitory effects of SM202190 or SP600125 remained (data not shown). Cell viability, as assessed by MTT assay, were not different between cell lines or altered by MAPK inhibitor, E_2_, or TGFβ treatment (data not shown).

Because ER and TGFβRII can each signal via shared non-canonical MAPK pathways, in addition to canonical effects on gene transcription via nuclear ER and TGFβ-stimulated Smad pathways, whose combined effects, for example, contribute to TGFβ-stimulated PTHrP secretion in bone-tropic ER- MDA-SA cells [24], the role of MAPK signaling was evaluated in E_2_ and/or TGFβ stimulated bone-tropic ER+ cells. MAPK signaling, as assessed by phosphorylation of second messengers p38, JNK1/2, and ERK1/2, was active at baseline in bone-tropic ER+ BMET-derived 43-4M cells (Figure 6A). E_2_ and TGFβ each further induced p38 and ERK activation at early (5 min) times, while only TGFβ (alone or in combination with E_2_) increased p38 and ERK activation at later (16 h) times (Figure 6A). Interestingly, at 10 min, ERK activation tended to transiently decrease in response to E_2_ and/or TGFβ (Figure 6A). Baseline JNK activation, in contrast, was minimally altered at any time in response to treatment with E_2_ and/or TGFβ (Figure 6A). Blockade of p38 or JNK activation by inhibitor pretreatment (SB2020190 or SP600125, respectively) appeared to partially block stimulated secretion of PTHrP by E_2_, TGFβ, or the combination (Figure 6B), while ERK inhibition (by SCH772984) was without effect (data not shown). However, after taking into account reductions in constitutive secretion by inhibitors of p38 or JNK (Figure 6B), no inhibitory effects remained (data not shown). Thus, MAPK pathways, while differentially activated by these two agents, had minimal, if any, role in mediating E_2_+/− TGFβ inducible PTHrP secretion in BMET-derived ER+ cells.

**Figure 7:**
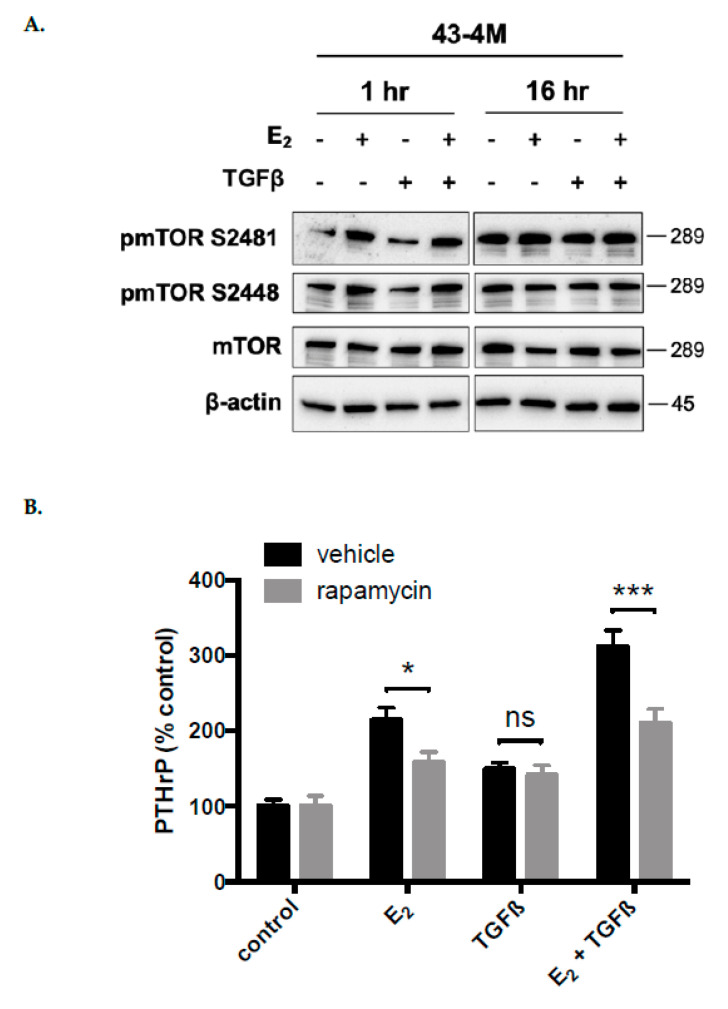
Effect of E_2_ and/or TGFβ on mTOR signaling. (**A**) mTOR expression and activation (phosphorylation of S2481 and S2448) in 43-4M cells treated concurrently with E_2_ and/or TGFβ for 1 or 16 h, following 4 days of E_2_ depletion; (**B**) inducible PTHrP secretion in 43-4M cells maintained for 4 days in E_2_-deplete media, then, treated for 52 h with E_2_ (10^−8^ M) and/or TGFβ (5 ng/mL), or media control (*n* = 4–8/group), with or without 1 h pretreatment with rapamycin (1 nM). Because rapamycin decreased constitutive PTHrP secretion by 46% (*p* < 0.05), data are expressed as % change relative to constitutive (control) levels. * *p ≤* 0.05 or *** *p* < 0.001 for rapamycin vs. vehicle with E_2_ and/or TGFβ treatment, by two-way ANOVA with Sidak’s post hoc test.

Because signaling via mTOR can facilitate nuclear ER signaling and is TGFβ-responsive [44,45,46,47], mTOR signaling was also assessed in E_2_ and/or TGFβ stimulated bone-tropic ER+ cells Signaling via mTOR, as assessed by S2481 and S2448 phosphorylation, was active at baseline in bone-tropic ER+ BMET-derived 43-4M cells (Figure 7A) and further stimulated by E_2_ at early (1 h) but not late times (16 h) (with or without TGFβ, Figure 7A), while TGFβ alone was without effect. Consistent with isolated activation of mTOR by E_2_, the mTOR inhibitor, rapamycin, significantly reduced PTHrP secretion stimulated by E_2_, alone or in combination with TGFβ, while stimulation of PTHrP by TGFβ alone was unchanged (Figure 7B). Thus, mTOR signaling appeared specific to E_2_ and contributed to E_2_ inducible PTHrP secretion in BMET-derived ER+ cells.

### 2.6. Role of TGFβ in Osteolytic ER+ BMET Progression In Vivo

The E_2_ dependence of tumor-associated osteolysis in ER+ BMETs formed in vivo by MCF-7 cells and ER-mediated secretion of PTHrP from these same cells, has previously been reported [12]. Given the correlation documented here between in vivo osteolytic ER+ BMET formation and (1) tumoral Smad-mediated TGFβ signaling, as well as (2) TGFβ-inducible tumoral PTHrP secretion, additive to the stimulatory effects of E_2_, a possible contributing role for TGFβ in driving osteolytic estrogen-dependent ER+ BMET progression in vivo was assessed. Using the same pan-TGFβ neutralizing antibody (1D11) and dosing scheme successfully used to block osteolytic BMET progression in preclinical models of ER- BMETs dependent on tumoral TGFβ signaling [30,31,32], experiments were undertaken to assess the in vivo effects of TGFβ neutralization on ER+ BMET progression in E_2_-treated nude mice inoculated with ER+ BMET-derived 43-4M cells, which secreted high levels of TGFβ-inducible PTHrP, alone or in combination with E_2_, unrelated to MAPK or mTOR signaling, suggesting a possible role for canonical Smad signaling in mediating the TGFβ effect.

**Figure 8:**
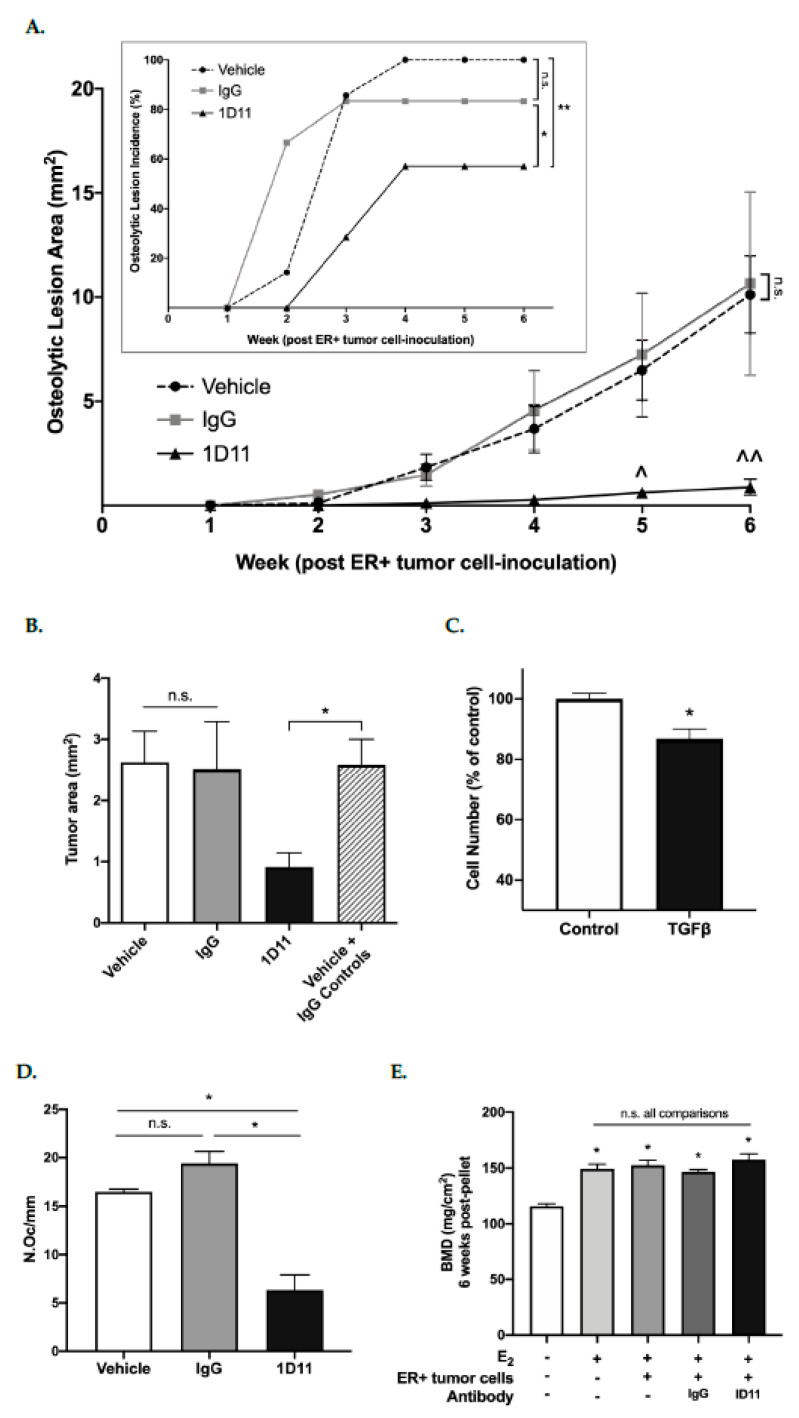
Effect of TGFβ inhibition on ER+ osteolytic BMET progression in vivo. (**A**) Osteolytic BMET lesion area and incidence (inset), as measured by radiographs and confirmed by histology, in hind limbs of E_2_-supplemented mice inoculated with 43-4M cells and treated 3 times/week for 6 weeks with TGFβ-neutralizing 1D11, isotype-matched control IgG (10 mg/kg), or vehicle (*n* = 6–7/group). ^ *p ≤* 0.05 and ^^ *p ≤* 0.001 1D11 vs. vehicle or IgG controls at indicated timepoints, n.s. IgG vs. vehicle, by two-way ANOVA with Tukey’s post-test; * *p ≤* 0.05 1D11 vs. IgG, ** *p ≤* 0.05 1D11 vs. vehicle, n.s. IgG vs. vehicle, by Gehan–Breslow–Wilcoxon test; (**B**) cytokeratin-positive 43-4M tumor area in hind limbs 6 weeks post-ER+ tumor cell inoculation in 1D11-treated mice vs. controls (IgG or vehicle-treated). Total tumor area per leg was unchanged by IgG treatment vs. vehicle (*n* = 7–13/group), therefore, values were combined and compared to 1D11-treated mice. * *p ≤* 0.05 by Student’s *t*-test; (**C**) effect of TGFβ (5 ng/mL) on 43-4M cell proliferation, as determined by MTT assay (*n* = 8/group), when cells were plated at a low density (6 × 10^4^ cells/2 cm^2^) to ensure treatments were added during the exponential growth phase. * *p ≤* 0.01 vs. media control by *t*-test; (**D**) osteoclast number at the tumor-bone interface (N.Oc/mm) in tibiae from E_2_-supplemented, 43-4M cell-inoculated mice treated with 1D11 vs. IgG or vehicle (*n* = 3–7/group). * *p ≤* 0.005 1D11 vs. vehicle or IgG, not significant (n.s.) vehicle vs. IgG, by one-way ANOVA with Tukey’s post hoc test; (**E**) areal bone mineral density of proximal femurs in naïve or E_2_ (0.72 mg)-supplemented mice 6 weeks following supplementation as compared with E_2_-supplemented mice inoculated with 43-4M cells and treated with 1D11 vs. IgG or vehicle 3 times/week for 6 weeks (*n* = 4–7/group). * *p ≤* 0.05 vs. control, with no significant differences (n.s.) between non-control groups by one-way ANOVA with Holm–Sidak’s post hoc test.

In the absence of E_2_ supplementation, osteolytic BMETs did not develop in mice inoculated with 43-4M cells (data not shown), but with E_2_ supplementation, osteolytic BMETs reached an incidence of 100% within 4 weeks of inoculation (Figure 8A inset, vehicle controls). Treatment with an IgG control antibody did not statistically alter osteolytic BMET incidence or size (Figure 8A and inset). Treatment with the pan-TGFβ neutralizing antibody (1D11) significantly reduced both osteolytic ER+ BMET lesion incidence and size in E_2_-supplemented mice as compared with vehicle or IgG controls (Figure 8A and inset), with the incidence decreased by 31% (*p ≤* 0.05) and lesion size by 92% (*p ≤* 0.0001) as compared with IgG control. TGFβ-neutralizing antibody treatment also decreased BMET tumor burden, as measured histologically by cytokeratin-positive tumor area, by 65% (*p* < 0.05) vs. combined control groups, although the trend comparing vehicle or IgG control groups individually to 1D11 did not reach statistical significance (Figure 8B). Of note, ER+ 43-4M cell proliferation was significantly inhibited by TGFβ treatment in vitro (Figure 8C), as has also been reported for bone-tropic ER- cells [22,31,32]. Thus, neutralization of TGFβ would be anticipated to increase tumor cell proliferation, which runs contrary to in vivo findings. Thus, reduced BMET size upon neutralization of TGFβ could not be attributable to direct TGFβ effects on tumor cell proliferation. Importantly, initiation of TGFβ neutralizing antibody treatment (24 h post inoculation) occurred after tumor dissemination to bone had already occurred and stabilized (e.g., 34.2 ± 7.5 vs. 36.7 ± 6.0 43-4M tumor cells per 10^6^ marrow cells, respectively, were detected in hind limbs 24 vs. 72 h post inoculation in vehicle-treated mice (*p* > 0.05, *n* = 4/group)). Lastly, because ER+ 43-4M cells did not form non-osseus metastases, effects of TGFβ neutralization on metastatic progression at other sites could not be assessed.

Consistent with the significant decrease in ER+ BMET-associated osteolysis documented with 1D11 treatment, osteoclast formation at the tumor-bone interface was also significantly reduced (65%) in mice treated with 1D11 (vs. controls) (Figure 8D). While inhibition of ER- BMET progression by 1D11 antibody treatment in mouse models lacking E_2_ supplementation is accompanied by significant anabolic effects of TGFβ neutralization on the bone microenvironment [30,32,48], areal bone mineral density (aBMD) of the proximal femur, a site devoid of ER+ BMETs in all treatment groups (data not shown), was not altered by TGFβ neutralization above levels already induced by E_2_ alone (Figure 8E), an effect that we have previously demonstrated to be attributable to enhanced anabolism [12,49].

## 3. Discussion

Despite the well-characterized function of tumoral TGFβ signaling in driving the “vicious cycle of osteolysis” demonstrated in preclinical models of ER- breast cancer BMETs, little is known about the role of tumoral TGFβ signaling in ER+ BMETs, a subtype that comprises over 70% of breast cancer BMETs [5,6,7,8]. TGFβ1 is expressed by bone myeloid cells [29,30,50,51,52,53], is the predominant TGFβ isoform in bone [54,55], and is released from bone matrix stores into the bone microenvironment during osteolysis, an event that dominates in the majority of breast cancer BMETs [56], regardless of the tumor subtype. Prior in vitro evidence has suggested that E_2_ could abate TGFβ signaling in breast cancer cells, and evidence of reciprocal expression of ERα and TGFβR in aggressive breast cancer cells has also been reported [34,35,37,38,39,40], suggesting that the emergence of TGFβ-targeted therapies for advanced stage cancers [21,33] could prove ineffective or less effective for patients with ER+ BMETs, which comprise the majority of breast cancer BMETs.

Contrary to this a priori hypothesis, in the studies presented here, TGFβ was in fact demonstrated to be a necessary driver of ER+ breast cancer osteolytic BMET progression in an E_2_-dependent ER+ model where tumor-associated osteolysis and osteolytic BMET progression are also dependent on tumoral ER signaling [12]. In vivo inhibition of microenvironmental TGFβ decreased ER+ tumor osteolytic progression and tumor burden, an effect unlikely to be due to changes in tumor cell dissemination, as treatment began 24 h post tumor inoculation after ER+ 43-4M cells had already disseminated to bone [12]; nor was the lower tumor burden upon TGFβ neutralization likely attributable to a loss of direct effects of TGFβ on tumor cell proliferation given TGFβ’s in vitro growth suppressive effects on luminal ER+ 43-4M cells, which are also consistent with anti-proliferative effects of TGFβ in bone-tropic ER- cells [22]. Instead, anti-osteolytic effects of TGFβ neutralization appear to be driving the reduction in osteolytic ER+ BMETs here, as in ER- models. The significant (65%) decrease in osteoclasts at the tumor-bone interface of ER+ BMETs observed in the 1D11 treatment group in parallel with profound (92%) inhibition of osteolytic lesion progression recapitulates results found in ER- osteolytic BMET preclinical models treated with the same TGFβ-neutralizing antibody used here [30,31,32]; TGFβR kinase inhibitors [30,31,32]; or with tumor-specific inhibition of Smad-mediated TGFβ signaling [22,27,28,57]. As previously noted, isolated blockade of TGFβ signaling in osteoclast precursors can also contribute to protective effects of TGFβ neutralization in osteolytic ER- BMET models where tumoral effects of TGFβ signaling are also operative, albeit with more modest and variable effects on osteolytic progression and osteoclast number as compared with selective inhibition of tumoral Smad-mediated TGFβ signaling [29,30,32]. Thus, it is possible that the anti-osteolytic effect of TGFβ neutralization documented here for ER+ BMETs may similarly be attributable to combined inhibition of tumoral and myeloid cell TGFβ signaling. However, running counter to this notion, in a BMET model using TGFβRII null ER+ MCF-7 variants lacking Smad signaling, which to our knowledge, is the only other study to examine a role for TGFβ in ER+ BMET, 1D11 antibody treatment did not alter progression of ER+ BMETs, which, interestingly, also had few tumor-associated osteoclasts, suggesting that myeloid TGFβ signaling may be insufficient to drive osteolytic BMET progression in the absence of tumoral TGFβ signaling [36].

The likely dependency of osteolysis and BMET progression in this E_2_-dependent ER+ model on tumoral TGFβ signaling ran counter to preexisting data suggesting possible suppression of TGFβ signaling in ER+ breast cancer cells. However, these in vivo results were in complete accord with additional key experimental findings, including the association of ER+ osteolytic bone metastatic behavior with TGFβ induction of canonical Smad signaling (MCF-7- and MCF-7J cells). While all ER+ cells expressed TGFβRII and TGFβRI at levels similar to those reported for ER- bone tropic cells, TGFβ-simulated Smad2/3 activation was lacking in cells that did not form E_2_-dependent osteolytic BMETs in vivo, due in part to reduced Smad3 expression in these cell lines. This suggestion of a potential Smad3 dependency for ER+ osteolytic BMET progression is interesting given prior similar evidence in bone-tropic ER- cells of a greater role for tumoral Smad3 (vs. Smad2) in TGFβ-induction of PTHrP or other key TGFβ-inducible target genes involved in osteolytic ER- BMETs, as well as slower in vivo osteolytic BMET progression following knock down of tumoral Smad3 expression [24,58]. In addition, clinical evidence presented here of co-expression of receptors initiating TGFβ and E_2_ signaling at higher levels in clinical breast cancer BMETs (vs. primary tumors) is also consistent with a key role of TGFβ signaling within the bone microenvironment for ER+ tumors, and with other clinical datasets associating TGFβRII expression with ER+ tumors [59]. Taken together, these findings suggest that TGFβ-stimulated Smad signaling, which is active in 75% of clinical BMET [29,60], may be a biomarker and/or necessary driver of osteolysis in ER+ BMETs, the absence of which may account, at least in part, for a lack of progression of osteolysis and tumor expansion in mice inoculated with T47D or ZR-75-1 cells, despite evidence here, and in prior studies, that these tumor cells disseminate to bone [61,62].

Because ER+ tumors have a greater clinical predilection for forming clinical BMETs, which are primarily osteolytic, our laboratory previously queried and documented a role for tumoral ERα signaling in specifically driving BMET osteolysis in the ER+ BMET models studied here, separate from its growth promoting proliferative effects, which are not bone specific [12]. An obvious question, thus, emerges, “In light of this new evidence of TGFβ dependence for both ER+ and ER- osteolytic BMETs, can the greater predilection of ER+ tumors for forming clinically evident, osteolytic BMETs be due to combined pro-osteolytic effects of tumoral ER and TGFβ signaling in ER+ breast cancer cells?” As demonstrated here, TGFβ stimulated Smad signaling was active and specifically associated with bone metastatic potential in ER+ cancer cells, with only transient E_2_-induced decreases in Smad2/3 activation, which were followed by later E_2_-induced increases in TGFβRII expression. Because TGFβ did not alter ER phosphorylation, a mechanism by which some growth factors after nuclear ER signaling [42], it is possible that additive effects of E_2_ and TGFβ on secretion of PTHrP, an osteolytic factor overexpressed in breast cancer BMETs vs. other metastatic sites [17,18] with similar overexpression here in ER+ BMET-derived cells, could simply be attributable to separate, but additive effects of canonical Smad-mediated TGFβRII and ER signaling (i.e., induction of gene expression by activated nuclear Smads and ERα). However, reports of activated Smad and ER each colocalizing with transcription factor, FOXA1, and/or direct interactions between Smad3 and ER [38] suggest that more complex interactions between canonical TGFβRII and nuclear ERα signaling may also drive the progression of osteolytic ER+ metastases in bone, a postulate that awaits further testing. While not studied here, it is also interesting to note that feedforward connections between ligands for these receptors are also possible in bone. While not relevant in E_2_-supplemented murine models that lack aromatase in bone [63,64], TGFβ-stimulated aromatase expression in human osteoblasts [65] could support local increases in E_2_ surrounding osteolytic ER+ BMETs, thus, further contributing to a vicious cycle tumor-driven osteolysis even in post-menopausal patients.

While MAPK and mTOR signaling were induced independently by E_2_ and/or by TGFβ, only E_2_ induction of mTOR signaling appears to partially mediate E_2_ induction of PTHrP. However, it remains possible that isolated or additive effects of E_2_ and/or TGFβ on these pathways could mediate other pro-metastatic events. The role of mTOR documented here for E_2_ stimulation of PTHrP secretion in bone tropic ER+ cells forming osteolytic lesions in vivo is specifically of clinical relevance as recent clinical trials evaluating the efficacy of combining hormone therapy with mTOR inhibitors (such as everolimus) have proven effective in patients with advanced ER+ breast cancer (including those with BMETs), with results reported here possibility providing additional mechanistic insights [66].

There are certain limitations to the studies reported here. Experiments were conducted using established human breast cancer cells lines rather than cells from patient-derived xenografts (PDX) models; while PDX models recapitulate aspects of patient-specific responses across a range of human tumors, ER+ PDXs are reported to have a much lower take rate and tend not to metastasize to bone [67,68,69]. Furthermore, the necessity of supplementing human xenograft ER+ BMET models with E_2_ to promote tumor growth and BMETs [12,61,70,71,72,73,74,75,76,77,78] causes notable anabolic changes in murine bone [49,61,72,79], which we previously demonstrated to have a stimulatory effect on ER+ BMETs [12], although these effects were independent of tumor-specific, pro-osteolytic effects of E_2_ that also drove ER+ BMET progression in the models described here [12]. However, from an experimental standpoint, this aspect of the ER+ models had the benefit of negating the anabolic effects of TGFβ inhibition on the bone microenvironment normally seen in naïve or ER- tumor-bearing mice [30,32,48]. While anabolic effects of TGFβ neutralization documented in ER- models lacking E_2_ supplementation have been postulated to contribute to the protective anti-osteolytic effects of TGFβ neutralization [32], because TGFβ neutralization had no such additive anabolic effect here, this can be discounted as a possible contributing factor to the therapeutic efficacy of TGFβ neutralization in the ER+ model.

While the presence, or absence, of TGFβ-stimulated Smad signaling and PTHrP secretion clearly differentiated ER+ cells that did, or did not, form osteolytic BMETs in vivo, it is likely that TGFβ signaling was not the only difference contributing to their differential abilities to form BMETs, and the involvement of other tumoral pathways and their interaction with the bone microenvironment waits further testing. However, the ability of in vivo TGFβ neutralization to significantly decrease ER+ BMETs by targeting osteolysis, and the demonstrated additive effects of ER and TGFβRII signaling in driving tumoral secretion of pro-osteolytic factors, such as PTHrP, clearly demonstrate, for the first time, a key role for TGFβ signaling in driving ER+ osteolytic BMET progression. The additive pro-osteolytic effects of tumoral TGFβRII and ER signaling in bone-tropic ER+ cells also suggest a possible mechanism underpinning the clinical observation that bone-disseminated ER+ (vs. ER-) breast cancer cells appear more likely to progress to clinically evident osteolytic BMETs. Further study of specific downstream molecular targets facilitating crosstalk between ER and TGFβRII in mediating osteolysis in bone-tropic ER+ cells, particularly in light of the high prevalence of activating ERα mutations in metastatic breast cancer [80,81] may, therefore, provide fertile ground for new therapeutic discoveries to benefit the majority of patients with breast cancer BMETs.

## 4. Materials and Methods

### 4.1. Cell Lines

Human ER+ breast cancer tumor cell lines, MCF-7, T47D, and ZR-75-1 (American Type Culture Collection (ATCC), Manassas, VA, USA), were cultured in E_2_-replete Dulbecco’s modified Eagle’s medium (DMEM, Invitrogen, Carlsbad, CA, USA) or RPMI-1640 (Invitrogen), as per ATCC’s recommendation, containing 10% heat-inactivated fetal bovine serum (FBS, Atlanta Biologicals, Flowery Branch, GA, USA), and 1% penicillin/streptomycin (Thermo Fisher, Waltham, MA, USA) in 37 °C and 5% CO_2_ in a humidified atmosphere. ATCC MCF-7 cells virally transduced to express firefly luciferase [73] (referred to as MCF-7J) were a kind gift provided by Dr. Jude Canon, and bone-tropic ER- human MDA-MB-231 (MDA-SA) cells [82] were generously provided by Dr. Theresa Guise. Authentication of all tumor cell lines was verified as previously described [41,43]. Where indicated, human ER+ BMET-derived tumor cells, isolated from hind limbs bearing radiographic evidence of osteolytic BMETs 42 to 56 days post-intracardiac (IC) inoculation, were also studied. To isolate cells, mice were euthanized, and tumor-bearing hind limbs were removed using sterile tools and stripped of connective tissue. Cells, flushed from the marrow of all tibiae and femurs of a single tumor-bearing animal using sterile media with repeated washing and crushing of bone, were combined, transferred into cell-culture dishes, and passaged when adherent tumor cells reached a confluency of 30% or higher. Tumor cells were passaged two additional times to remove non-immortalized and non-adherent murine cells before establishment of human cell lines (43-4M, 41-1M, 41-2M, 38-2M, 56M, or 61M from MCF-7 cells, or 84-2MJ from MCF-7J cells), which were each authenticated as being MCF-7-derived before use [41,43]. Of note, cell proliferation in E_2_-replete media over 8 days of incubation, determined by counting of trypsinized cells, was statistically the same in MCF-7 vs. BMET-derived MCF-7 cells, with cell numbers increasing approximately 6-fold.

### 4.2. In Vitro Analysis of ER and TGFβ Signaling in ER+ Human Breast Cancer Cells

For analysis of effects of E_2_ and/or TGFβ on cell signaling by Western, cells were first maintained in E_2_-deplete media (phenol red-free DMEM, 10% charcoal-stripped FBS, 200 mM l-glutamine, 1% penicillin/streptomycin) for 4 days, as previously described [12], prior to treatment for the indicated times with 17β-estradiol (E_2_, 10^−8^ M, Sigma-Aldrich, St. Louis, MO, USA) and/or TGFβ (TGFβ1, 5 ng/mL, R&D Systems, Minneapolis, MN, USA), with treatments either added concurrently or with E_2_ (vs. vehicle) pretreatment for 1–23 h, as indicated, prior to direct addition of TGFβ. For Western analyses of protein levels and/or activation (phosphorylation), proteins were isolated from whole cell lysates, quantified by Bradford assay (Bio-Rad, Hercules, CA, USA), and analyzed by Western blots with confirmation of even protein loading, as previously described, including assessment of β-actin [41,43]. Blots were probed with primary rabbit antibodies shown in Supplemental Table S1, followed by HRP-conjugated anti-rabbit secondary antibody (#7074, Cell Signaling Technology [CST], Danvers, MA, USA) and chemiluminescent visualization of SuperSignal West Femto ECL substrate (ThermoFisher). Prestained Protein Marker (#13953, CST, Danvers, MA, USA), or Biotinylated Protein Ladder followed by anti-biotin HRP-linked secondary antibody (#7727, CST, Danvers, MA, USA), were used to estimate probed-proteins ≤190 kDa in molecular weight, and Precision Plus Protein Standard (#161-0374, Bio-Rad, Hercules, CA, USA) followed by anti-biotin HRP-linked secondary antibody (#7727, CST, Danvers, MA, USA) was used for probed-proteins ≥190 kDa. Most blots are representative of ≥3 separate experiments.

For analysis of the tumor-secreted osteolytic factor, PTHrP, cells were plated in 24-well tissue culture plates at a density of 1.3 × 10^5^ cells/well and maintained in E_2_-deplete media for 4 days followed by treatments with E_2_ (10^−7^ or 10^−8^ M, as indicated) and/or TGFβ (5 ng/mL), or media control for 48, 52, or 72 h, as indicated (depending on cell line) to optimize detection. For inhibitor experiments, cells were pretreated for 1 h prior to E_2_ and/or TGFβ stimulation with MAPK inhibitors and doses previously demonstrated to block TGFβ-inducible PTHrP secretion from ER- MDA-SA cells [24], using p38 inhibitor SB202190 [10 µM], JNK inhibitor SP600125 [25 µM], ERK inhibitor SCH772984 (1 nM) (Selleckchem, Houston, TX, USA), or with a rapamycin to block mTOR signaling (1 nM, #9904, CST, Danvers, MA, USA), using a dose that does not alter MCF-7 cell proliferation [83] (and data not shown). Conditioned media, stored at −80 °C after addition of protease inhibitors (Sigma-Aldrich, St. Louis, MO USA), were assayed for secreted PTHrP using a commercial immunoradiometric assay (sensitivity 10–14 pg/mL, Beckman Coulter, Brea, CA, USA). Cell numbers, determined by counting of trypsinized cells, remained statistically unchanged (from day 0) after 4 days of E_2_ depletion for every cell line, and a lack of treatment effect under the conditions of the experiments on cell number during the incubation periods for all reported values was also verified using a commercial MTT assay (ATCC, Manassas, VA, USA).

The effects of TGFβ (4 ng/mL, for 4 days) on cell proliferation vs. E_2_-replete media alone were assessed separately in cells plated at a low density (6 × 10^4^ cells/well in a 24-well tissue culture plate) with subsequent analysis of cell number by MTT assay (ATCC, Manassas, VA, USA), as per the manufacturer’s protocol.

### 4.3. RNA Gene Expression Clinical Primary Breast Tumor vs. BMET-Derived Tumor Cell

Publicly available RNA expression data (GSE39494) were analyzed to compare ERα vs. TGFβRII expression in primary breast cancer tumors from women who developed BMETs vs. breast cancer cells isolated from bones with clinical evidence of metastases. For this dataset [84], RNA isolated from primary breast tumors (*n* = 5) vs. laser captured and micro-dissected tumor cells from flash frozen trephine bone biopsies of non-matched breast cancer patients with BMETs (*n* = 5), as well as universal human reference RNA, were amplified and conjugated to Cy3 dye prior to hybridization using an Agilent whole human genome microarray platform (Agilent, Santa Clara, CA, USA). Raw expression data from GEO data GSE39494 were analyzed using R scripts and Bioconductor modules. The arrays were normalized using normexp background correction and quantile normalization. When multiple probes existed for a gene, these data were averaged. Log2 normalized data were further assessed for differential expression using Limma [85] analysis, which includes correction for multiple hypothesis testing by a false discovery rate method. An adjusted *p*-value ≤0.05 was considered to be significant. For choosing relevant genes a combination of fold change (2-fold up or down) and significant *p*-values were chosen. A R gplots library was utilized to generate heatmaps and plots.

### 4.4. Animal Studies

All animal protocols were approved by the Institutional Animal Care and Use Committee at The University of Arizona (protocol # 08-149, 9 March 2018) in accordance with the National Institutes of Health Guide for the Care and Use of Laboratory Animals. Four-week-old female *Foxn1^nu^* athymic nude outbred mice were purchased from Envigo (Indianapolis, IN, USA) and housed in plastic cages in laminar flow isolated hoods with access to water and autoclaved mouse chow ad libitum. Mice (*n* = 5–13/group) were inoculated at 5 weeks of age with 1 × 10^5^ ER+ tumor cells via the left cardiac ventricle (intracardiac, IC) three days post placement of 60-day extended release 0.72 mg 17β-estradiol (E_2_) pellets (Innovative Research of America, Sarasota, FL, USA), as previously described [12,41,49]. In one experiment, similarly E_2_-supplemented mice inoculated with BMET-derived 43-4M tumor cells (*n* = 5–7/group) were treated for 6 weeks with a pan-TGFβ-neutralizing murine monoclonal antibody (clone 1D11.16.8, #BE0057, BioXCell, Lebanon, NH, USA) vs. isotype-matched murine control IgG (M0PC-21 clone, #BE0083, BioXCell, Lebanon, NH, USA) or vehicle alone (in vivo Pure pH 7.0 dilution buffer, #IP0070, BioXCell, Lebanon, NH, USA). The TGFβ neutralizing antibody and dosing scheme (10 mg/kg IP, 3 times/week) matched those previously successfully used to prevent ER- BMET progression [30,31,32] in preclinical models. Antibody dosing began 24 h post inoculation, when 43-4M tumor cells had already disseminated to bone, as was confirmed in a separate experiment, where inoculated Vybrant DiD-labelled 43-4M tumor cells in E_2_-pelleted mice were isolated from proximal tibias and quantitated as previously described 24 or 72 h post inoculation [12]. No changes in health status occurred requiring euthanasia in tumor-cell inoculated mice, which were also examined at gross necropsy (or via bioluminescence for MCF-7J cells) 6 weeks post inoculation for non-osseus metastases.

### 4.5. Histologic Assessment of ER+ BMETs

Epithelial ER+ breast cancer tumors were immunohistochemically identified in midsagittal (depth of 400–500 um) sections (5–6 µm thick) of decalcified, formalin fixed, paraffin-embedded hind leg bones using primary antibodies to pan-cytokeratin (#Z0622, Agilent Dako, Santa Clara, CA, USA) or human ERα (#ab108398, Abcam, Cambridge, UK), with tumor area in hind limbs measured in a blinded fashion (and expressed per leg), as previously described [12,41]. Hematoxylin and eosin (H&E) stained sections were also used to assess tumor morphology in bone. Multinucleated tartrate-resistant acid phosphatase (TRAP)-positive osteoclasts lining metaphyseal bone surfaces were quantified at the tumor-bone interface of BMET-bearing mice, as previously described [12,41], and are reported as osteoclast number per mm of tumor-bone interface [12,41,86].

### 4.6. Bone Imaging

Osteolytic lesion formation was assessed via weekly radiographs of mouse hind limbs (Faxitron UltraFocus 1000, Faxitron Bioptics, Tucson, AZ, USA) in E_2_-supplemented ER+ tumor cell-inoculated mice over the 6-week course of experiments, which was analyzed as previously described in a blinded fashion by three independent investigators using ImageJ software (NIH) [12,41], with osteolytic BMET incidence or total hind limb radiographic osteolytic lesion area reported per mouse, including animals without osteolytic lesions. Because E_2_ can induce osteolytic osteosarcoma formation in nude mice [49], osteolytic breast cancer BMETs in each hind limb bone were verified by either correlating radiographic lesions with cytokeratin-positive human tumors [49,72], or bioluminescence (BLI, Lago, Spectral Instruments Imaging, Tucson, AZ, USA) in the case of mice inoculated with luciferase transfected MCF-7J cells following IP injection with 150 mg/kg of 15 mg/mL d-luciferin potassium salt (#LUCK, Gold Biotechnology, St. Louis, MO, USA) dissolved in sterile PBS. Areal bone mineral density (aBMD) of proximal femurs (25%), which were devoid of osteolytic BMETs in tumor-inoculated mice, was assessed by dual-energy X-ray absorptiometry (DXA, Faxitron UltraFocus 1000) in mice 6 weeks post E_2_-supplementation, with or without tumor inoculation and additional treatments, as indicated.

### 4.7. Statistical Analysis

Data are reported as mean ± SEM, with statistical significance of two-sided *p*-values defined as *p* ≤ 0.05. Statistical differences were determined using Prism 8.0 software (Graphpad, San Diego, CA, USA) for *t*-tests, one- or two-way analyses of variance (ANOVA) with post hoc testing (as indicated), and Gehan–Breslow–Wilcoxon tests. For experiments determining inhibitor effects on E_2_- and/or TGFβ-stimulated PTHrP secretion, statistical effects were determined by two-way ANOVA for absolute values and, when inhibitors significantly reduced constitutive secretion (*p* < 0.01), by *t*-test for E_2_- and/or TGFβ-stimulated increases over constitutive levels in inhibitor-treated (vs. control) cells.

## Data Availability

A publicly available dataset was analyzed in this study. These data can be found here: https://www.ncbi.nlm.nih.gov/geo/query/acc.cgi?acc=GSE39494 (accessed on 19 March 2021). Other data presented in this study are available upon request from the corresponding author.

## Supplementary Materials

The following are available online at https://www.mdpi.com/article/10.3390/ijms22094463/s1.
