# Supplementary material for: A Role for TGFβ Signaling in Preclinical Osteolytic Estrogen Receptor-Positive Breast Cancer Bone Metastases Progression
Source: Int J Mol Sci. 2021 Apr 24;22(9):4463. doi: 10.3390/ijms22094463 (PMC8123146; doi:10.3390/ijms22094463)
Supplement: Supplementary file 1 [file ijms-22-04463-s001.zip › ijms-1170993-supplementary.pdf]

SUPPLEMENTAL TABLES AND FIGURES

Supplemental Table S1. Primary antibodies used for Western blots.

Antigen	Manufacturer	Catalog #
ERα	Cell Signaling Technology (CST)	8644
pERα S104/106	Abcam	ab75753
pERα S118	Abcam	ab32396
TGFβRI	CST	3712
TGFβRII	CST	3713[1]
TGFβRII	CST	79424
pSMAD2	CST	3108
SMAD2	CST	5339
pSMAD3	CST	9520
SMAD3	CST	9523
SMAD4	CST	38454
pp38	CST	4511
p38	CST	8690
pERK1/2	CST	4370
ERK1/2	CST	4695
pJNK1/2	CST	4668
JNK1/2	CST	9252
pmTOR S2481	CST	2974
pmTOR S2448	CST	2971
mTOR	CST	2972
E-cadherin	Abcam	ab15148
Vimentin	CST	5741
β-actin	CST	4967

[1]This antibody was used in Figure S1A.

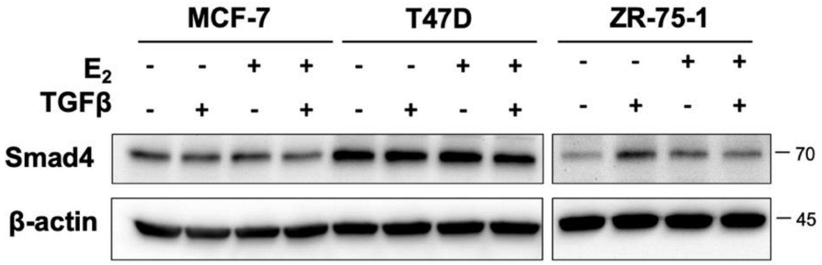

Supplemental Figure S1. Expression of Smad4 in ER+ breast cancer cells.
Smad4 expression in MCF-7, T47D, and ZR-75-1 maintained in E_2-deplete media for four days prior to E_2 (10^{-8} M) stimulation (23 hours), followed by 1 hour of TGFβ (5 ng/ml).

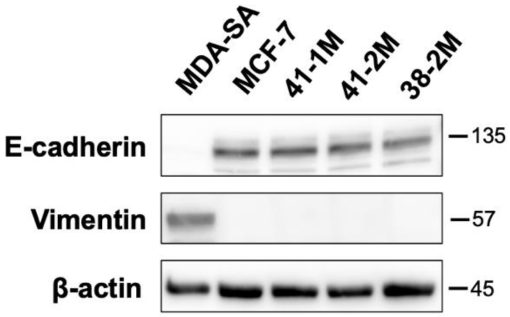

Supplemental Figure S2. ER+ BMET-derived cells maintain parental phenotype.
E-cadherin vs. vimentin expression in ER+ MCF-7, T47D, ZR-75-1, and/or MCF-7 BMET-derived cells (38-2M, 41-1M, 41-2M and/or 43-4M), as compared to ER- MDA-SA cells by Western blot analysis.
